# Biodegradation of Crude Oil by Nitrate-Reducing, Sulfate-Reducing, and Methanogenic Microbial Communities under High-Pressure Conditions

**DOI:** 10.3390/microorganisms12081543

**Published:** 2024-07-27

**Authors:** Lu Wang, Yong Nie, Xinglong Chen, Jinbo Xu, Zemin Ji, Wenfeng Song, Xiaofang Wei, Xinmin Song, Xiao-Lei Wu

**Affiliations:** 1 State Key Laboratory of Enhanced Oil & Gas Recovery, Beijing 100083, China; luwangmoon@petrochina.com.cn (L.W.); chxlhdpu@petrochina.com.cn (X.C.); jizemin@petrochina.com.cn (Z.J.); xiaofangwei@petrochina.com.cn (X.W.); 2 Research Institute of Petroleum Exploration & Development, Beijing 100083, China; songwf@petrochina.com.cn; 3 College of Engineering, Peking University, Beijing 100083, China; nieyong@pku.edu.cn (Y.N.); 1801110631@pku.edu.cn (J.X.); 4 Institute of Ecology, Peking University, Beijing 100083, China

**Keywords:** oil reservoir, anaerobic biodegradation, pressure, nitrate-reducing, sulfate-reducing, methanogenesis

## Abstract

Carbon capture, utilization, and storage (CCUS) is an important component in many national net-zero strategies, and ensuring that CO_2_ can be safely and economically stored in geological systems is critical. Recent discoveries have shown that microbial processes (e.g., methanogenesis) can modify fluid composition and fluid dynamics within the storage reservoir. Oil reservoirs are under high pressure, but the influence of pressure on the petroleum microbial community has been previously overlooked. To better understand microbial community dynamics in deep oil reservoirs, we designed an experiment to examine the effect of high pressure (12 megapascals [MPa], 60 °C) on nitrate-reducing, sulfate-reducing, and methanogenic enrichment cultures. Cultures were exposed to these conditions for 90 d and compared with a control exposed to atmospheric pressure (0.1 MPa, 60 °C). The degradation characteristic oil compounds were confirmed by thin-layer analysis of oil SARA (saturates, aromatics, resins, and asphaltenes) family component rods. We found that the asphaltene component in crude oil was biodegraded under high pressure, but the concentration of asphaltenes increased under atmospheric pressure. Gas chromatography analyses of saturates showed that short-chain saturates (C8–C12) were biodegraded under high and atmospheric pressure, especially in the methanogenic enrichment culture under high pressure (the ratio of change was −81%), resulting in an increased relative abundance of medium- and long-chain saturates. In the nitrate-reducing and sulfate-reducing enrichment cultures, long-chain saturates (C22–C32) were biodegraded in cultures exposed to high-pressure and anaerobic conditions, with a ratio of change of −8.0% and −2.3%, respectively. However, the relative proportion of long-chain saturates (C22–C32) increased under atmospheric pressure. Gas Chromatography Mass Spectrometry analyses of aromatics showed that several naphthalene series compounds (naphthalene, C1-naphthalene, and C2-naphthalene) were biodegraded in the sulfate-reducing enrichment under both atmospheric pressure and high pressure. Our study has discerned the linkages between the biodegradation characteristics of crude oil and pressures, which is important for the future application of bioenergy with CCUS (bio-CCUS).

## 1. Introduction

The increase in atmospheric greenhouse gas concentrations, primarily caused by the use of fossil fuels, has created serious risks for humanity [1]. Carbon capture, utilization, and storage (CCUS) is one way to achieve low-carbon use of high-carbon fuels, and it is recognized as an important technology package for climate change mitigation [2]. CCUS is expected to reduce emissions by approximately 600–1400 Mt CO_2_ by 2050 and by 1000–1800 Mt CO_2_ by 2060. One-third of this reduction is expected to result from the use of bioenergy with CCUS by 2060 [3,4]. CO_2_ can be stored in oil reservoirs and change the mobility and future trapping systematics of the evolved supercritical fluid. Recent discoveries have shown that microbial processes (e.g., methanogenesis) may modify the fluid composition and fluid dynamics within a storage reservoir and could reduce the volume of injected CO_2_ required [5]. In subsurface deposits, CO_2_ utilization and crude oil biodegradation is coupled with the microbial community [6,7,8,9,10]. Recently, researchers discovered that stochastic assembly processes are critical in shaping the groundwater microbial community structure; however, the relative importance of these processes decreased as environmental stress increased [11]. In the subsurface, environmental stressors on the microbial community include temperature [12], salinity [13,14], pH [15], water content [16], electron acceptors [17,18,19,20,21], and the composition of crude oil [15,22]. Oil reservoirs are extreme environments with high temperature and high pressure [23], with all reservoir fluids existing under pressure. High pressure is a key characteristic of oil reservoirs, but its influence on the microbial community has been previously overlooked.

Pressure exists in a reservoir for the same reason that pressure exists at the bottom of the ocean. The Deepwater Horizon oil spill was one of the largest and deepest oil spills recorded, and it has been shown that pressure (0.1, 15, and 30 MPa) acts synergistically with low temperature to slow microbial growth and oil degradation in deep-sea environments [24]. Pressure has also been reported to restructure deep-sea hydrocarbon-degrading microbial communities [23,25,26]. Using a high-temperature and high-pressure incubation system (55 °C, 5 MPa), microbial communities capable of methanogenic crude oil degradation were obtained from the Yabase oil reservoir [27] and Yamagata oil reservoir [28] in Japan. Mild hydrostatic pressure (15 MPa) shaped the assemblage of oil-degrading communities [29]. Recently, petroleum-degrading microbial communities incubated under high-pressure conditions (12 MPa) were metabolically profiled with metagenomics, which also defined microbial community interactions and the exchange of amino acids and cofactors among members [23]. In addition, pressurized anaerobic digestion has gained increasing interest in recent years. It is a valuable process that allows the production of biogas with high methane content, reducing the energy costs for the biogas to upgrade and inject into the distribution grid. A modified Anaerobic Model Digestion n.1 showed that the higher the pressure, the higher the volumetric mass transfer coefficient [30]. The research results of Merkle et al. [31] showed that methane content had increased from 79.08% at 10 bar to 90.45% at 50 bar. Siciliano’s research showed that as the pressure increased, the quality of the biogas was enhanced, while the overall amount of methane lowered [32].

These studies enriched our current knowledge of microbial communities in petroleum reservoirs under high-pressure conditions. 

Biodegradation in shallow subsurface petroleum reservoirs has been attributed to aerobic bacterial hydrocarbon degradation stimulated by surface recharge of oxygen-bearing meteoric waters. However, anaerobic degradation processes dominate in subsurface sedimentary environments [33]. Approximately half of the world’s in-place oil and bitumen has experienced biodegradation, which is believed to largely have occurred through anaerobic methanogenesis. The presence of secondary microbial methane is apparent in twenty-two basins, probable in twelve basins, and possible in six basins worldwide [34]. Therefore, research on anaerobic degradation of oil is important. Crude oil can contain thousands or even tens of thousands of hydrocarbon compounds with highly variable composition [35,36]. Hydrocarbons are divided into four parts based on their polarizability and polarity (saturates, aromatics, resins, and asphaltenes; SARA), with each fraction having a different composition [37]. Saturated hydrocarbons are more easily degraded than aromatic hydrocarbons. Aromatic hydrocarbons with one to three aromatic rings are also efficiently biodegraded. The asphaltene fraction contains higher-molecular-weight compounds with complex chemical structures in the range of 600 to 2,000,000 Da [38,39]. 

In the subsurface, the oil biodegradation rate is not limited by the supply of electron donors (i.e., hydrocarbons) but rather by the supply of nutrients or electron acceptors to the site of degradation [40]. For hydrocarbon or polycyclic aromatic hydrocarbon (PAH) degradation, NO_3_^−^, Fe^3+^, SO_4_^2−^, and HCO_3_^−^ are typical terminal electron acceptors (TEAs), which are linked to four typical reducing conditions (i.e., nitrate-reducing, ferric-reducing, sulfate-reducing, and methanogenic conditions, respectively) [18,41,42,43,44]. The standard Gibbs free energy values for NO_3_^−^, SO_4_^2−^, and CO_2_ are −163.2, −152.2, and −62.8 kJ·mol^−1^, respectively [45]. Previous studies have indicated that the biodegradation of PAHs and the mechanisms affecting bioremediation in PAH-polluted marine sediment may vary under different TEA-reducing conditions [44,46]. Chen et al. [47] reviewed the recent advances in the biodegradation of PAHs under anoxic conditions and provided mechanistic insights into metabolic pathways and functional genes. Fumarate addition is also an important initial activation mechanism for anaerobic alkane degradation and methanogenic alkane degradation, resulting in the generation of 1-methylalkyl succinic acids; this is followed by the C-skeleton rearrangement reaction, oxidization to fatty acids, and further conversion to methane and carbon dioxide [48,49,50,51]. In the presence of nitrate, CO_2_ can improve the anaerobic biodegradation efficiency of the resins and asphaltenes in heavy oil, particularly the biodegradation selectivity of polar heterocyclic compounds by the newly isolated *Klebsiella michiganensis* [52]. However, there have been limited studies on anaerobic biodegradation using different electron acceptors under high-pressure conditions. 

In the present study, we conducted laboratory-scale high-pressure incubations of production water from Jilin Oilfield under nitrate-reducing, sulfate-reducing, and methanogenic conditions. We detected and analyzed the composition of crude oil after incubation. The study aimed to characterize the role of anaerobic biodegradation at pressure under various conditions.

## 2. Materials and Methods

### 2.1. High-Pressure and Atmospheric-Pressure Incubations

**Figure 1:**
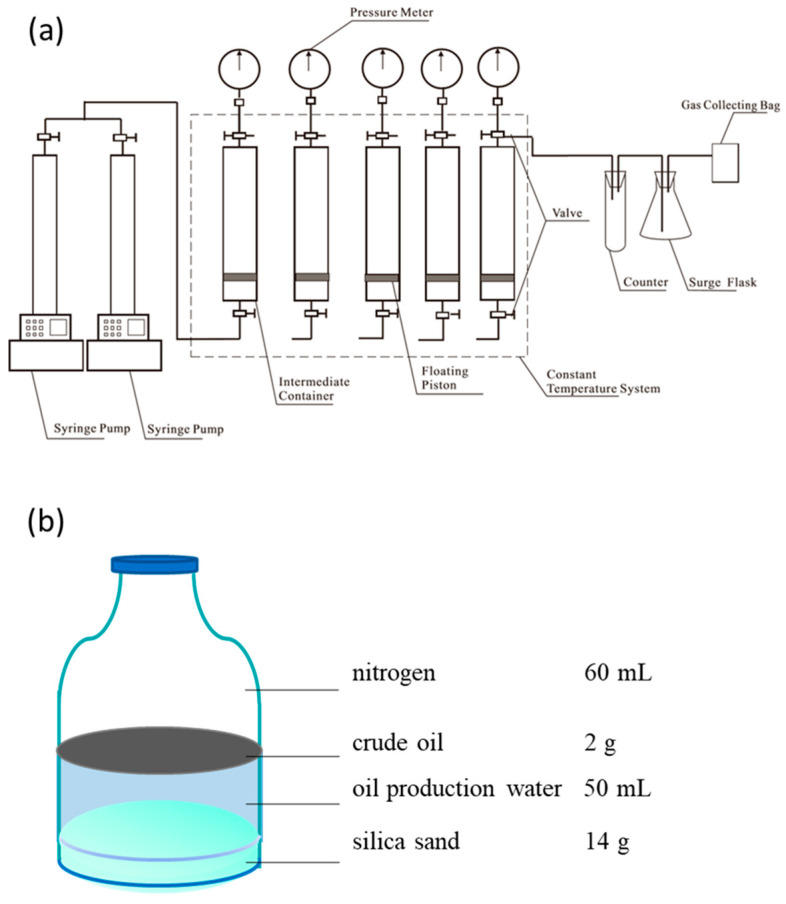
The setup diagram of experiment. (**a**) high-pressure group; (**b**) atmospheric pressure group.

Crude oil and production water were collected from Jilin Oilfield in China. The temperature of the oil reservoir was 60 °C. After the oil production well was selected, dead oil was discarded before sampling. Culture vessels (or the pressure container) were quickly filled with oil production water and sealed, and then transported to the local research institute. Solution gases were released, and the vessels were replenished with oil production water. The pressure of the samples was increased and they were stored in stainless steel reactors (1 L) at 5 MPa. The samples were transported to the laboratory within 48 h, at which point the pressure was immediately increased to 12 MPa. For the three stainless steel reactors (1 L), the first one was added by fumarate (final concentration of 10 mM), the second by nitrate (final concentration of 10 mM), and the third by sulfate (final concentration of 10 mM). All of the three high-pressure reactors were incubated statically for 90 days at 60 °C. The setup diagram is shown in Figure 1a. Samples that were intended to be cultured at atmospheric pressure were transported from Jilin Oilfield to the laboratory under atmospheric pressure. Our atmospheric pressure setup is the same as the one for conventional anaerobic experiments using Hungate technology. The setup diagram at atmospheric pressure is shown in Figure 1b. Hungate serum bottles with a total volume of 120 mL were cleaned and dried. We filled a bottle with 14 g of 40–70 mesh quartz sand (about 10 mL in volume) (Tianjin Quartz Clock Factory Bazhou Chemical Plant, analytically pure), and placed it in 180 °C oven for 3 h for sterilization. We turned off the oven and waited until the temperature dropped below 60 °C. After having opened the door of the oven, we used tin foil to seal the bottle immediately. We filled the bottle with 50 mL of oil production water (incubation broth) and 2 g of crude oil, and the headspace volume was 60 mL. Fumarate, nitrate, and sulfate were added to each of the three groups cultured under atmospheric pressure, respectively. The final concentration of additives in each group was the same, 10 mM. After having added indicator resazurin into the sample bottles, the anaerobic reduction copper column and air nitrogen replacement device were used to inject high-purity nitrogen through the injection needles for about 15–20 min until the color of the liquid in the bottles changed from red to colorless. Finally, the mixed solution of L-Cysteine HCl·H_2_O (3 mM) and Na_2_S·9H_2_O (2 mM) was quickly added into each serum bottle. We sealed these bottles immediately with a rubber plug (SANSHIN, Okinawa, Japan) and finally with an aluminum seal cap (Chemglass, Vineland, NJ, USA). Then, these bottles were incubated statically for 90 d at 60 °C.

The culture starting point sample was labeled S0. After 90 days of incubation, the samples incubated under atmospheric pressure were labeled as “additive-90”, e.g., NO_3_-90, SO_4_-90, and Fuma-90; the samples incubated under high pressure were labeled as “additive-P90”, e.g., NO_3_-P90, SO_4_-P90, and Fuma-P90. 

After 90 days of incubation, the total microbial DNA was extracted from the incubation water, and the bacteria were amplified (16S rRNA gene), purified, and sequenced using the HiSeq platform. For each sample, 200–250 mL of water sample in the culture system was collected using a filter membrane. The filter membrane was cut into 1 mm^2^ fragments and transferred to a Lysing Matrix E tube with glass beads. Amounts of 978 μL Sodium Phosphate Buffer and 122 μL MT Buffer were added. After homogenization, the bacteria were sonicated at 16 °C for 30 min, and the genomic DNA of the bacteria was extracted according to the instructions of the FastDNA^®^ Spin Kit for soil kit (MP Biomedicals, Santa Ana, CA, USA). High-throughput sequencing of the 16S rRNA gene was conducted by Beijing Novogene Biotech Co., Ltd. (Beijing, China). The abundance of Operational Taxonomic Units (OTUs for short) at the level of the genus was labeled “g-”. The OTUs of samples incubated for 90 d under atmospheric pressure were labeled as “g-additive-90” (“-1” and “-2” stand for two parallel samples), e.g., g-NO_3_-90-1, g-NO_3_-90-2, g-SO_4_-90-1, g-SO_4_-90-2, g-Fuma-90-1, and g-Fuma-90-2. (g-NO_3_-90 means the average value of g-NO_3_-90-1 and g-NO_3_-90-2, etc.).

The OTUs of samples incubated for 90 d under high pressure were labeled as “g-additive-P90”, e.g., g-NO_3_-P90, g-SO_4_-P90, and g-Fuma-P90. 

After 90 days of incubation, the change ratio of each *n*-alkane relative to 0 d equals the ratio of C_i_ at 90 d (C_i-90_) in a sample minus the ratio of C_i_ at 0 d (C_i-0_), divided by (C_i-0_), i.e., ratio of change = (C_i-90_ − C_i-0_) × 100%/C_i-0._

### 2.2. Analysis of the Family Composition of Crude Oil

A group-type analysis can be used to define the relationship between the structures and properties of an oil because it is impossible to identify all of the individual components in oil [53]. The procedure to separate the family composition into saturates, aromatics, resins, and asphaltenes (SARA procedure) was conducted in accordance with the oil and gas industry standard of the People’s Republic of China SY/T 5119-2016 [54], which is an analysis method for the family composition of rock extracts and crude oil. Testing was carried out by the Central Laboratory of Geological Sciences, Research Institute of Petroleum Exploration & Development, PetroChina. 

The steps of using the rod thin layer flame ionization detection method to conduct analysis are as follows: (a) Take a specific amount of soluble organic matter from the rock or purified crude oil sample, dissolve it in chloroform, and prepare a solution with a concentration ranging from 10 mg/mL to 20 mg/mL. (b) Extract 0.5 μL to 1.0 μL of the sample solution using a microsyringe and apply it to an activated silica gel chromatographic rod, approximately 0.5 cm from one end, and repeat this process five–six times. (c) Place the silica gel chromatographic rod in a constant humidity cylinder for 10 min. (d) Insert the silica gel chromatographic rod into a chromatographic cylinder filled with *n*-hexane and unfold it so that the solvent rises between 8 cm and 9 cm. (e) Allow the silica gel chromatographic rod to sit in volatile solvent at room temperature for 2 min before placing it back in the constant humidity cylinder for another 10 min. (f) Subsequently, place it into a chromatographic cylinder containing a mixed solvent of dichloromethane and *n*-hexane (volume ratio of 1:1), unfold it until the solvent rises between 4 cm and 5 cm. (g) Repeat step e), successively. Finally, insert it into a chromatographic cylinder containing a mixture of *n*-hexane and isoamyl alcohol (volume ratio of 90:10); unfold it until the solvent rises between 1.5 cm and 2.0 cm. The separated sample on the silica gel chromatographic rod should be placed in volatile solvent at room temperature for 2 min. (h) Turn on the main power switch and other switches of the rod thin layer flame ionization analyzer, and adjust the instrument parameters according to the regulations (room temperature: 20 °C–30 °C; relative humidity: less than or equals to 65%; airflow rate: 2000 mL/min; hydrogen flow rate: 160 mL/min–180 mL/min; and scanning speed: 30 s each time). The separated sample on the silica gel thin layer rod should be inserted into the instrument for testing.

### 2.3. Gas Chromatography Analysis of Saturates

The procedure to separate saturated hydrocarbon was conducted in accordance with the oil and gas industry standard of the People’s Republic of China SY/T 5779-2008 [55], which is an analytical method for hydrocarbons in petroleum and sediment by gas chromatography. Testing was conducted by the Central Laboratory of Geological Sciences, Research Institute of Petroleum Exploration & Development, PetroChina. The test method was based on Part 6 of *SY/T 5779-2008: Analysis of saturated hydrocarbons in rock chloroform extracts and crude oil*. 1. Sample preparation. (1) The sample should be fractionated according to SY/T 5119 to isolate the saturated hydrocarbon fractions, which should then be concentrated and transferred into a sealed sample bottle for storage in a refrigerated environment prior to testing. (2) When a splitter is used during sampling, the saturated hydrocarbon should be diluted with an appropriate amount of *n*-hexane; when sampling without a splitter, the saturated hydrocarbon should be diluted with an appropriate amount of isooctane. 2. Measurement procedure. (1) Activate the gas path system of the chromatograph to eliminate any blockages or leaks. (2) Initiate the instrument following the specific operating procedures for different types of chromatographs and adjust it to achieve optimal operating conditions. (3) Ignite the flame; after having stabilized the programmed temperature’s chromatographic baseline, select either the shunt or non-shunt injection method, and determine the injection volume based on the sample size. (4) Inject the sample using a microinjector, and activate both the programmed temperature settings and chromatographic workstation.

### 2.4. Gas Chromatography Mass Spectrometry Analysis of Aromatics

The aromatic hydrocarbon samples were analyzed using the Thermo-Trace GC U1tra-DSQ II (Model: 0807173, made in Thermo Fisher Scientific, Waltham, MA, USA). The oven was heated to 100 °C for 5 min, and the temperature was increased at 3 °C/min until it reached 320 °C and then held for 20 min. To avoid the influence of a solvent peak, the filament was opened after 8 min. The separation column was an HP-5MS elastic quartz capillary column (60 m × 0.25 mm × 0.25 mm). High-purity nitrogen (99.999%) was used as a carrier gas at a flow rate of 1 mL/min. The injector temperature was 300 °C and the transmission line temperature was 300 °C.

The mass spectrometer was operated in the electron impact mode using 70 eV ionization voltage. The ion source temperature was 220 °C and the GC/MS interface was set to 250 °C. Testing was conducted by the Central Laboratory of Geological Sciences, Research Institute of Petroleum Exploration & Development, PetroChina.

## 3. Results

The effects of high pressure and atmospheric pressure on the biodegradation of crude oil were compared based on the results of family composition, saturated hydrocarbon chromatography, and aromatic hydrocarbon mass spectrometry. 

### 3.1. Nitrate-Reducing Enrichment Group

#### 3.1.1. Family Composition of Crude Oil

**Figure 2:**
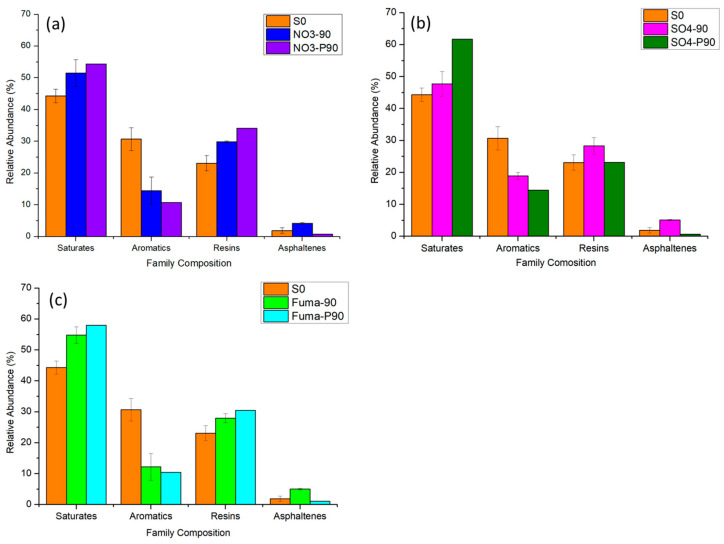
Relative abundance of family composition of different groups. (**a**) S0, NO_3_-90, and NO_3_-P90; (**b**) S0, SO_4_-90, and SO_4_-P90; and (**c**) S0, Fuma-90, and Fuma-P90. (The culture starting point sample was labeled S0. After 90 days of incubation, the samples incubated under atmospheric pressure were labeled as “additive-90”, e.g., NO_3_-90, SO_4_-90, and Fuma-90, while the samples incubated under high pressure were labeled as “additive-P90”, e.g., NO_3_-P90, SO_4_-P90, and Fuma-P90).

The family composition of the atmospheric pressure group and the high-pressure group at 0 d and after 90 d of nitrate-reducing cultivation is shown in Figure 2a. The relative abundance of saturates increased after 90 d of cultivation by 7.2% at atmospheric pressure and 10.0% at high pressure. The relative abundance of aromatics at atmospheric pressure and high pressure decreased by 16.2% and 20.0%, respectively, after 90 d cultivation. The relative abundance of resins at atmospheric pressure and high pressure increased by 6.8% and 11.1%, respectively, after 90 d cultivation. Compared with that at 0 d, the relative abundance of asphaltenes in the atmospheric pressure group was 2.3% higher after 90 d and was 1.1% lower in the high-pressure group.

#### 3.1.2. Gas Chromatography of Saturates

**Figure 3:**
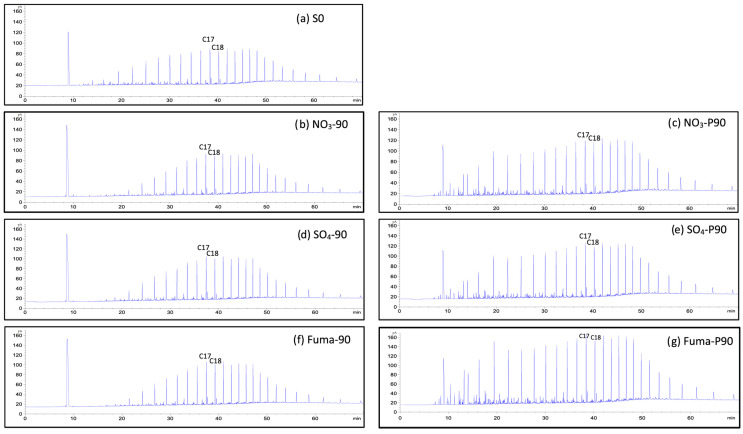
Gas chromatography of saturates before and after microbial degradation of crude oil. (**a**) S0, (**b**) NO_3_-90, (**c**) NO_3_-P90, (**d**) SO_4_-90, (**e**) SO_4_-P90, (**f**) Fuma-90, and (**g**) Fuma-P90. (The culture starting point sample was labeled S0. After 90 days of incubation, the samples incubated under atmospheric pressure were labeled as “additive-90”, e.g., NO_3_-90, SO_4_-90, and Fuma-90, while the samples incubated under high pressure were labeled as “additive-P90”, e.g., NO_3_-P90, SO_4_-P90, and Fuma-P90).

**Figure 4:**
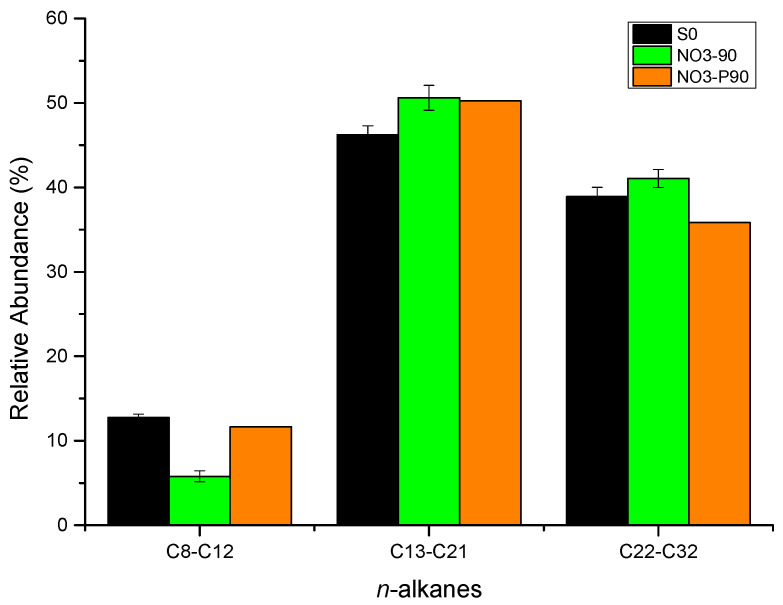
Relative abundance of *n*-alkanes from C8–C12, C13–C21, and C22–C32. (The culture starting point sample was labeled S0. After 90 days of incubation, the samples with NO_3_^−^ additive incubated under atmospheric and high pressure were labeled as NO_3_-90 and NO_3_-P90, respectively).

The gas chromatography of saturates before and after microbial degradation of crude oil with nitrate is shown in Figure 3a–c and Figure S1. For short-chain alkanes (C8–C12), the relative abundance decreased by 7.0% after incubation at atmospheric pressure and 1.1% at high pressure after 90 d. For medium-chain alkanes (C13–C21), the relative abundance increased by 4.4% and 4.0% after incubation for 90 d at atmospheric and high pressure, respectively. For long-chain alkanes (C22–C32), the relative abundance increased by 2.1% after incubation under atmospheric pressure and decreased by 3.1% after incubation under high pressure (Figure 4 and Figure S2). These results indicate that the microbial community tends to degrade long-chain alkanes under high pressure but not under atmospheric pressure.

#### 3.1.3. Gas Chromatography and Mass Spectrometry of Aromatics

The results from the gas chromatography and mass spectrometry of aromatics before and after the microbial degradation of crude oil with nitrate are shown in Figure S5a–c. From these mass spectrograms, it is difficult to see a clear difference.

### 3.2. Sulfate-Reducing Enrichment Group

#### 3.2.1. Family Composition of Crude Oil

The hydrocarbon family compositions of the atmospheric pressure group and the high-pressure group at 0 d and after 90 d of sulfate-reducing cultivation are shown in the Figure 2b. The relative abundance of saturates was 3.4% higher after 90 d at atmospheric pressure and 17.4% higher after 90 d at high pressure. The relative abundance of aromatics was 11.8% lower after 90 d at atmospheric pressure and 16.3% lower after 90 d at high pressure. The relative abundance of resins after 90 d was 5.2% lower at atmospheric pressure and 0.1% lower at high pressure. The relative abundance of asphaltenes was 3.2% higher after 90 d at atmospheric pressure and 1.2% lower after 90 d at high pressure.

#### 3.2.2. Gas Chromatography of Saturates

**Figure 5:**
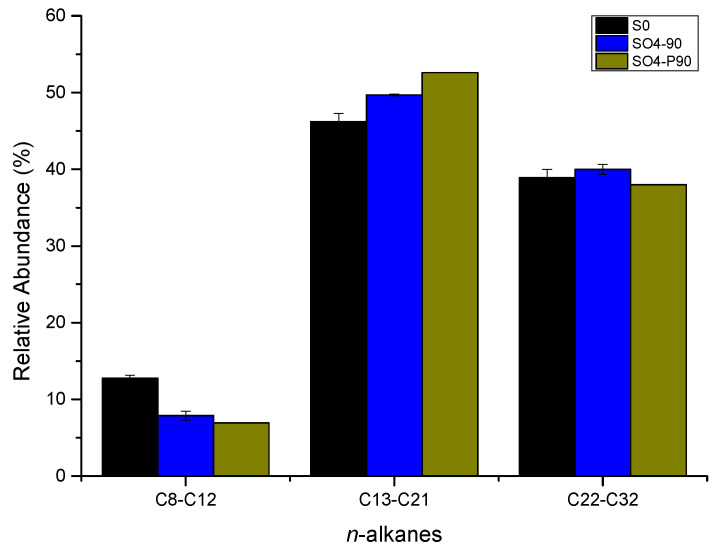
Relative abundance of *n*-alkanes from C8–C12, C13–C21, and C22–C32. (The culture starting point sample was labeled S0. After 90 days of incubation, the samples with SO_4_^−^ additive incubated under atmospheric and high pressure were labeled as SO_4_-90 and SO_4_-P90, respectively).

The gas chromatography of saturates before and after microbial degradation of crude oil with sulfate reduction is shown in Figure S5a,d,e. For short-chain alkanes (C8–C12), the relative abundance decreased by 4.9% after incubation at atmospheric pressure and by 5.8% at high pressure. For medium-chain alkanes (C13–C21), the relative abundance increased by 3.5% after incubation at atmospheric pressure and by 6.4% after incubation at high pressure. For long-chain alkanes (C22–C32), the relative abundance increased by 1.1% after incubation at atmospheric pressure and decreased by 0.9% after incubation at high pressure (Figure 5 and Figure S3). The results indicate that the microbial community tends to degrade long-chain alkanes at high pressure but not significantly under atmospheric pressure.

#### 3.2.3. Gas Chromatography and Mass Spectrometry of Aromatics

**Figure 6:**
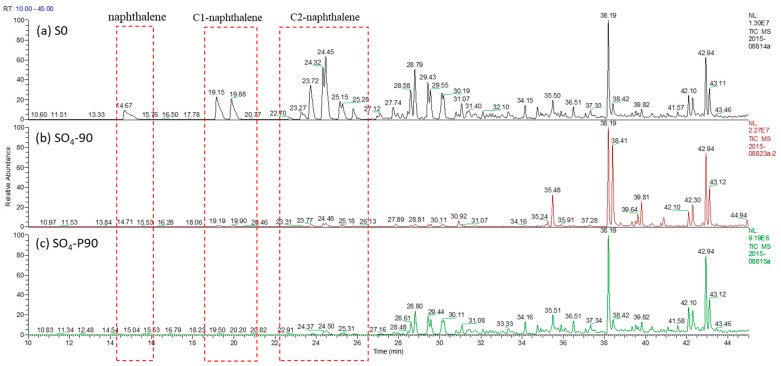
Total Ion Chromatography Mass Spectrometry of aromatic hydrocarbons of different samples (retention time is shown from 10 min to 45 min). (**a**) S0. (**b**) SO_4_-90. (**c**) SO_4_-P90. (The culture starting point sample was labeled S0. After 90 days of incubation, the samples with SO_4_^−^ additive incubated under atmospheric and high pressure were labeled as SO_4_-90 and SO_4_-P90, respectively).

The results of the gas chromatography and mass spectrometry for aromatics before and after the microbial degradation of crude oil by sulfate are depicted in Figure S5a,d,e. For a comprehensive comparison of microbial degradation, Figure 6 illustrates the Total Ion Chromatography Mass Spectrometry results for aromatics over a duration ranging from 10 to 45 min. Naphthalene, C1-naphthalene, and C2-naphthalene have undergone biodegradation through sulfate reduction enrichment culture.

### 3.3. Methanogenic Enrichment Group

#### 3.3.1. Family Composition of Crude Oil

The family compositions of hydrocarbons from the atmospheric pressure group and the high-pressure group at 0 d and after 90 d of methanogenic cultivation are shown in Figure 2c. The relative abundance of saturates was 10.5% higher at atmospheric pressure and 13.7% higher at high pressure after 90 d. The relative abundance of aromatics was 18.5% lower at atmospheric pressure and 20.3% lower at high pressure after 90 d. The relative abundance of resins at atmospheric pressure was 4.9% higher and 7.4% higher at high pressure after 90 d. The relative abundance of asphaltenes was 3.1% higher in the atmospheric pressure group and 0.8% lower in the high-pressure group.

#### 3.3.2. Gas Chromatography of Saturates

**Figure 7:**
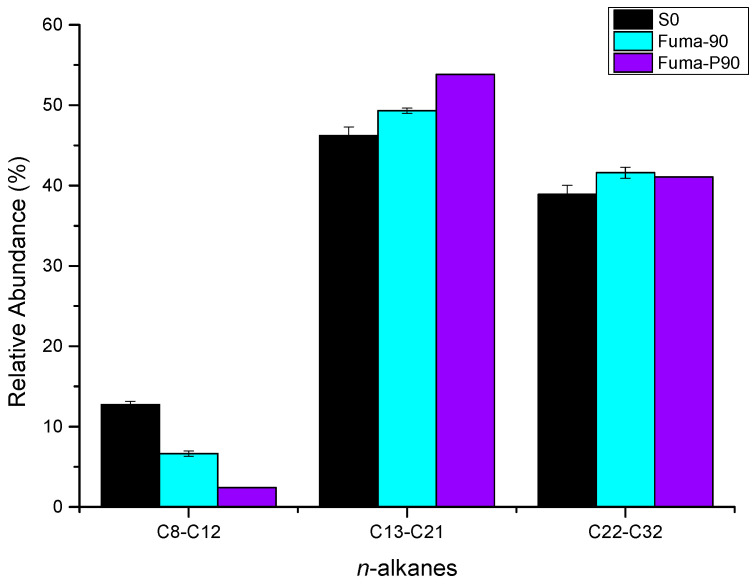
Relative abundance of *n*-alkanes from C8–C12, C13–C21, and C22–C32. (The culture starting point sample was labeled S0. After 90 days of incubation, the samples with fumarate additive incubated under atmospheric and high pressure were labeled as Fuma-90 and Fuma-P90, respectively).

The results from the gas chromatography of saturates before and after the microbial degradation of crude oil with sulfate are shown in Figure 3a,f,g and Figure S1. For short-chain alkanes (C8–C12), the relative abundance was 6.1% lower after incubation at atmospheric pressure and 10.34% lower after incubation at high pressure. For medium-chain alkanes (C13–C21), the relative abundance increased by 3.1% after incubation at atmospheric pressure and by 7.6% after incubation at high pressure. For long-chain alkanes (C22–C32), the relative abundance increased by 2.7% after incubation at atmospheric pressure and by 2.2% after incubation at high pressure. These results indicate that, comparing the degradation of medium- and long-chain alkanes, the microbial community tends to degrade short-chain alkanes under high pressure as well as under atmospheric pressure (Figure 7 and Figure S4).

#### 3.3.3. Gas Chromatography and Mass Spectrometry of Aromatics

The results from the gas chromatography and mass spectrometry of aromatics before and after the microbial degradation of crude oil with nitrate are presented in Figure S5a,f,g. From these mass spectrograms, it is difficult to see a clear difference. 

## 4. Discussion

The results from the atmospheric pressure and high-pressure incubation experiments confirm that the microbial anaerobic degradation of crude oil occurred during the 90 d incubation. However, hydrocarbon biodegradation ability under atmospheric pressure and high pressure was different.

### 4.1. Effect of the Biodegradation Efficiency of Oil Family

After 90 d of incubation, aromatics decreased in both the atmospheric and high-pressure groups for the nitrate-reducing, sulfate-reducing, and methanogenic enrichment cultures; the degree of decrease in the high-pressure culture group was higher than that for the atmospheric-pressure culture group. Saturates and resins increased in both the atmospheric- and high-pressure groups, except for the resins under sulfate-reducing conditions and high pressure, which increased by 0.1%. Interestingly, asphaltenes increased in the atmospheric pressure group and decreased in the high-pressure group. This indicates that asphaltenes tend to be degraded by the microbial community under high pressure. Asphaltenes are the most polar and heavy fraction of petroleum and have complex structures and toxicity [56]. Only a few microbial consortia have been reported to degrade asphaltenes. These consortia contain isolates that correspond to the genera *Rhodococcus*, *Bacillus*, *Stutzerimonas*, *Cellulosimicrobium*, *Pseudomonas*, and *Paenibacillus*, which are able to use asphaltene as a sole carbon and energy source [15,56,57]. *Bacillus*, *Pseudomonas*, and *Paenibacillus* genera were detected in the high-pressure group in this study (Table S1, sheet 1 and sheet 2). We speculated that these piezotolerant microorganisms had survived in the oil reservoir for a long time, were adapted to the high-pressure environment, and had the ability to use asphaltenes. Their ability to withstand high temperature and degrade aromatics and asphaltenes led to an increase in the saturates, subsequently improving the quality of the oil.

### 4.2. Effect of Oil Saturate Hydrocarbon Biodegradation Efficiency

**Figure 8:**
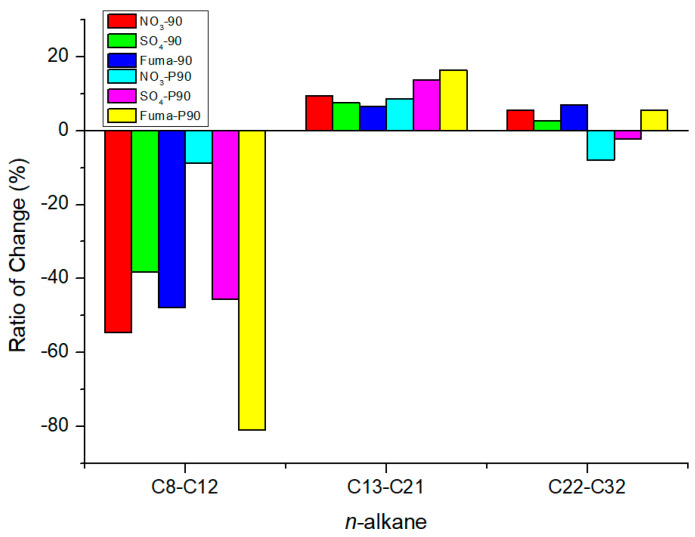
The ratio of change for *n*-alkanes from C8–C12, C13–C21, and C22–C32. (After 90 days of incubation, the samples incubated under atmospheric pressure were labeled as “additive-90”, e.g., NO_3_-90, SO_4_-90, and Fuma-90, while the samples incubated under high pressure were labeled as “additive-P90”, e.g., NO_3_-P90, SO_4_-P90, and Fuma-P90).

**Figure 9:**
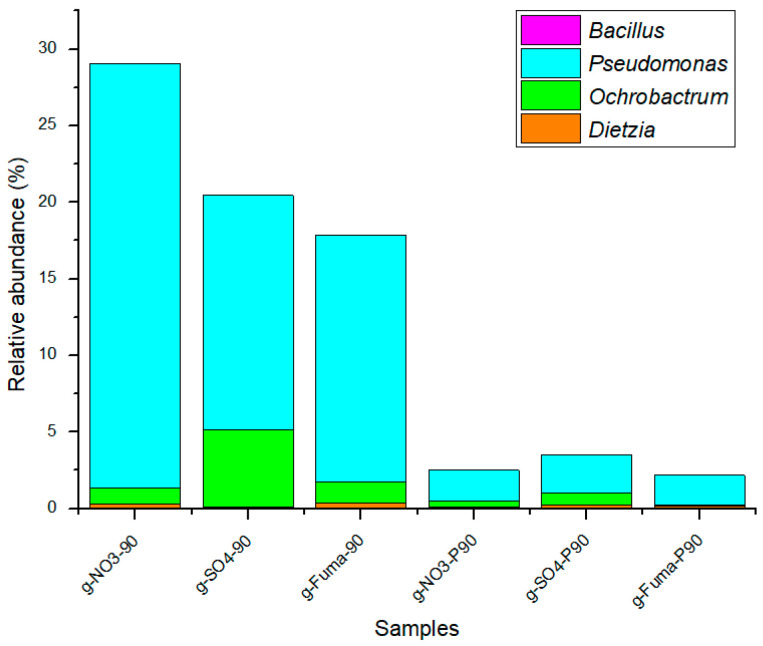
The composition and relative abundance of the genera *Bacillus*, *Pseudomonas*, *Ochrobactrum,* and *Dietzia* in different samples.

After 90 d of incubation, the ratio of change (see Section 2.1) is shown in Figure 8. For the nitrate-reducing, sulfate-reducing, and methanogenic enrichment cultures, the *n*-alkanes C8–C12 decreased in both the atmospheric and high-pressure groups, while the *n*-alkanes C13–C21 increased in each group. The *n*-alkanes C22–C32 decreased in each group under atmospheric pressure; however, the changes in the relative abundance of this hydrocarbon fraction in each group were different under high-pressure cultivation. When nitrate and sulfate were used as electron acceptors at high pressure, the relative abundance of C22–C32 decreased. In the methanogenic enrichment cultures at high pressure, the relative abundance of the C22–C32 fraction increased by 2.2% and the ratio of change was 5.6%, while the relative abundance of the C8–C12 fraction decreased by 10.3% with a ratio of change was −81.0%. In this set of experiments, the relative abundance of short-chain alkanes may have decreased, resulting in an increase in the relative abundance of medium- and long-chain alkanes. Short-chain saturates tended to be degraded in both the atmospheric- and high-pressure groups, and long-chain saturates tended to be degraded at high pressure. Previous studies have reported the degradation of hydrocarbons by the genus *Dietzia*. *Dietzia* sp. DQ12-45-1b used C6–C40 *n*-alkanes as carbon and energy sources [58]; *Dietzia* sp. E1 used C6–C36 alkanes [59]; *D. cinnamea* P4 used C11–C36 alkanes [60]; and *Dietzia maris* AURCCBT01 used C14, C18, C20, C28, and C32 alkanes [61]. Consortia with dominant *Geobacillus*, *Parageobacillus*, and *Anoxybacillus* genera have exhibited a strong ability to degrade hydrocarbons in long-chain alkanes (C18–C40) [62]. The consortia of *Ochrobactrum* sp., *Pseudomonas aeruginosa*, and *Bacillus* sp. have the ability to use C11–C18 alkanes, with a removal rate up to 78.5%, and the removal rates of C26–C29 and C33–C35 alkanes were 36.2% and 30.5%, respectively. In addition, possible metabolic pathways of crude oil degradation were also proposed by this study, including reactions for aldehyde bond (H-C=O) and ketone bond (C-O-C) hydrodehydration, ring-opening of cyclic ether, C-C bond oxidation of benzene rings, and ring-opening hydrolysis of gentisic acid [63]. *Rhodococcus qingshengii* and *Alcanivorax venustensis* incubated in a seawater-based medium degraded 100% of C9–C12 and C16–C29 hydrocarbons and 85% of C13–C15 hydrocarbons [64]. The biodegradation of crude oil paraffin wax was demonstrated by 11 bacteria (including the genera *Geobacillus*, *Parageobacillus*, and *Anoxybacillus*) isolated from seawater and oil-contaminated soil samples; these bacteria could completely degrade C37–C40 alkanes and increase the ratio of C14–C18. In addition, enzymes associated with the biodegradation of crude oil, including alkane monooxygenase, alcohol dehydrogenase, lipase, and esterase, were detected [65]. In our study, the genera *Dietzia*, *Ochrobactrum*, *Pseudomonas*, and *Bacillus* were detected based on 16S rRNA, and their relative abundances are shown in Figure 9. Although the total relative abundance of the oil-biodegrading microorganisms incubated at atmospheric pressure was higher than that of those incubated at high pressure, the oil-degrading ability of the former was not significantly better than the latter. High pressure and high temperature in the in situ oil reservoir may be important for the function of microbial communities.

### 4.3. Effect of Oil Aromatic Hydrocarbon Biodegradation Efficiency

**Figure 10:**
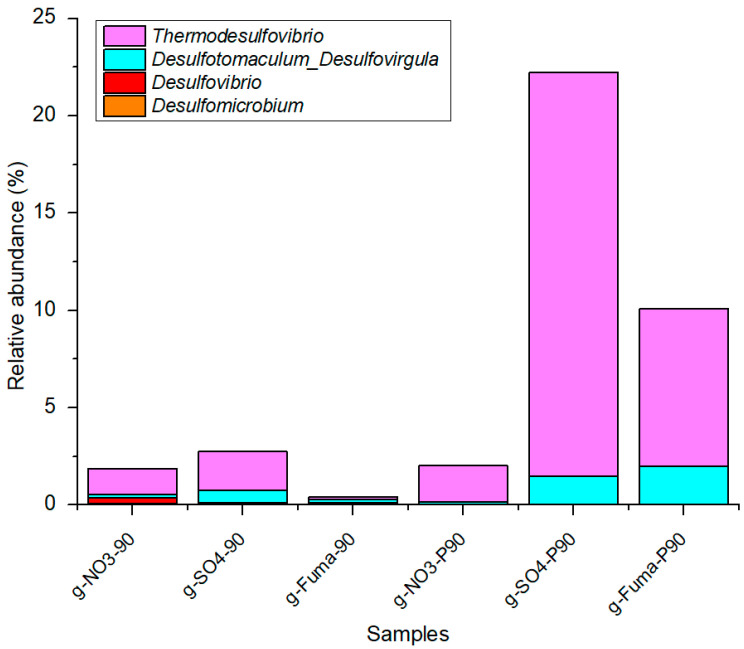
The composition and relative abundance of the genera *Thermodesulfovibrio*, *Desulfotomaculum_Desulfovirgula*, *Desulfovibrio,* and *Desulfomicrobium* in different samples.

From the results of the Total Ion Chromatography Mass Spectrometry of aromatic hydrocarbons, degradation under atmospheric pressure and high pressure was significant when using sulfate as an electron acceptor. In Figure 6, naphthalene, C1-naphthalene (including 2-methylnaphthalene and 1-methylnaphthalene), and C2-naphthalene (including 2-ethylnaphthalene, 2,6-dimethylnaphthalene, 2,7-dimethylnaphthalene, 1,3-dimethylnaphthalene, 1,7-dimethylnaphthalene, 1,6-dimethylnaphthalene, 1,4-dimethylnaphthalene, 2,3-dimethylnaphthalene, and 1,2-dimethylnaphthalene) were largely degraded. Chen et al. [47] reported that several naphthalene-degrading consortia can use bicyclic PAHs (i.e., naphthalene) as a sole carbon source, mainly under sulfate-reducing conditions. Figure 10 presents the sulfate-reducing bacteria found in our study, including *Desulfomicrobium*, *Desulfovibrio*, *Desulfotomaculum_Desulfovirgula*, and *Thermodesulfovibrio* (Table S1, sheet1 and sheet 3). The abundance of sequences related to the genus *Thermodesulfovibrio* was 20.8% and 2.0%, respectively, under high and atmospheric pressure with sulfate as an electron acceptor. The ability of these genera to withstand high temperature and degrade bicyclic PAHs results in an improvement in the quality of the oil.

## 5. Conclusions

In summary, we measured the change in oil composition to determine the microbial hydrocarbon degradation ability under high pressure in nitrate-reducing, sulfate-reducing, and methanogenic enrichment cultures. In the nitrate-reducing and sulfate-reducing enrichment cultures, the relative abundance of asphaltenes in the atmospheric pressure group was higher after 90 d incubation than at 0 d, while the asphaltenes in the high-pressure group decreased over the 90 d of incubation. The microbial community tended to degrade long-chain alkanes (C22–C32) under high pressure but not under atmospheric pressure. In the sulfate-reducing enrichment, the degradation of aromatics under atmospheric pressure and high pressure was significant. In the methanogenic enrichments, the relative abundance of asphaltenes in the atmospheric pressure group was higher after 90 d incubation, and was lower after 90 d at high pressure. The relative abundance of the C8–C12 alkane fraction decreased by a great degree under high-pressure incubation. This study has enriched our current knowledge of the oil-degradation profile of microbial communities under high-temperature and high-pressure conditions.

## Data Availability

The original contributions presented in the study are included in the article, further inquiries can be directed to the corresponding authors.

## Supplementary Materials

The following supporting information can be downloaded at: https://www.mdpi.com/article/10.3390/microorganisms12081543/s1. Table S1. Composition and relative abundance of different genera in all samples; Figure S1. Gas chromatography of saturates before and after microbial degradation of crude oil; Figure S2. Relative abundance of *n*-alkanes from C8–C32 with NO_3_^−^ additive; Figure S3. Relative abundance of *n*-alkanes from C8–C32 with SO_4_^2−^ additive; Figure S4. Relative abundance of n-alkanes from C8–C32 with fumarate additive; Figure S5. Total Ion Chromatography Mass Spectrometry of aromatic hydrocarbon from 7 min to 60 min.
