# Supplementary material for: Biodegradation of Crude Oil by Nitrate-Reducing, Sulfate-Reducing, and Methanogenic Microbial Communities under High-Pressure Conditions
Source: Microorganisms. 2024 Jul 27;12(8):1543. doi: 10.3390/microorganisms12081543 (PMC11356252; doi:10.3390/microorganisms12081543)
Supplement: Supplementary file 1 [file microorganisms-12-01543-s001.zip › microorganisms-3084745-Supplementary Materials Figures.pdf]

Supplementary Materials:

Figure S1. Gas chromatography of saturates before and after microbial degradation of crude oil. (a)S0, (b)NO_3-90, (c)NO_3-P90, (d)SO_4-90, (e)SO_4-P90, (f)Fuma-90, (g)Fuma-P90. (The culture starting point sample was labeled S0. After 90 days of incubation, the samples incubated under atmospheric pressure were labeled as "additive-90", e.g., NO_3-90, SO_4-90 and Fuma-90, while the samples incubated under high pressure were labeled as "additive-P90", e.g., NO_3-P90, SO_4-P90 and Fuma-P90.)

Figure S2. Relative abundance of *n*-alkanes from C8-C32 with NO_3^- additive. (The culture starting point sample was labeled S0. After 90 days of incubation, the samples with NO_3^- additive incubated under atmospheric and high pressure were labeled as NO_3-90 and NO_3-P90, respectively.)

Figure S3. Relative abundance of *n*-alkanes from C8-C32 with SO_4^{2-} additive. (The culture starting point sample was labeled S0. After 90 days of incubation, the samples with SO_4^{2-} additive incubated under atmospheric and high pressure were labeled as SO_4-90 and SO_4-P90, respectively.)

Figure S4. Relative abundance of *n*-alkanes from C8-C32 with fumarate additive. (The culture starting point sample was labeled S0. After 90 days of incubation, the samples with fumarate additive incubated under atmospheric and high pressure were labeled as Fuma-90 and Fuma-P90, respectively.)

Figure S5. Total Ion Chromatography Mass Spectrometry of aromatic hydrocarbon (retention time has been showed from 7 min to 60 min. (a)S0, (b)NO_3-90, (c)NO_3-P90, (d)SO_4-90, (e)SO_4-P90, (f)Fuma-90, (g)Fuma-P90. (The culture starting point sample was labeled S0. After 90 days of incubation, the samples incubated under atmospheric pressure were labeled as "additive-90", e.g., NO_3-90, SO_4-90 and Fuma-90, while the samples incubated under high pressure were labeled as "additive-P90", e.g., NO_3-P90, SO_4-P90 and Fuma-P90.)

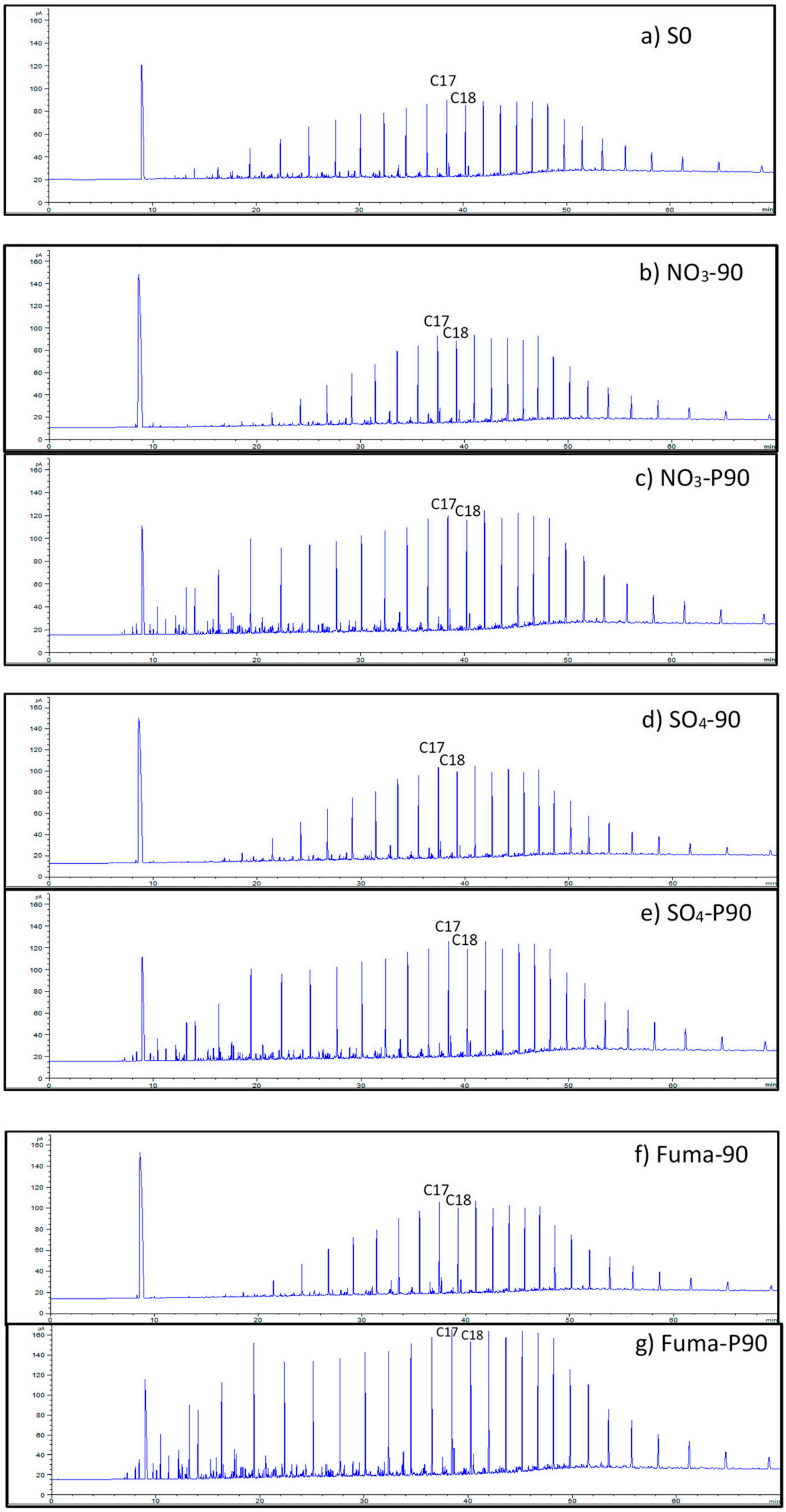
a) S0
C17
C18
b) NO_3-90
C17
C18
c) NO_3-P90
C17
C18
d) SO_4-90
C17
C18
e) SO_4-P90
C17
C18
f) Fuma-90
C17
C18
g) Fuma-P90
C17
C18

Figure S1

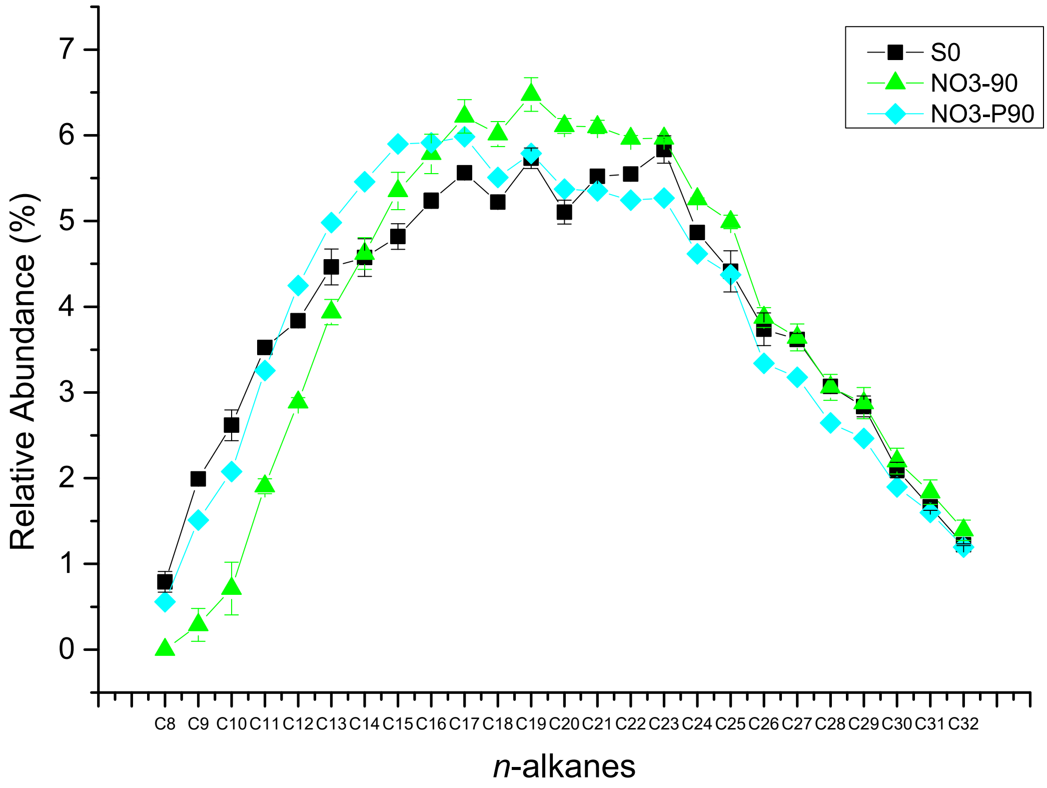

Relative Abundance (%)
n-alkanes
S0
NO3-90
NO3-P90
C8 C9 C10 C11 C12 C13 C14 C15 C16 C17 C18 C19 C20 C21 C22 C23 C24 C25 C26 C27 C28 C29 C30 C31 C32
0 1 2 3 4 5 6 7

Figure S2

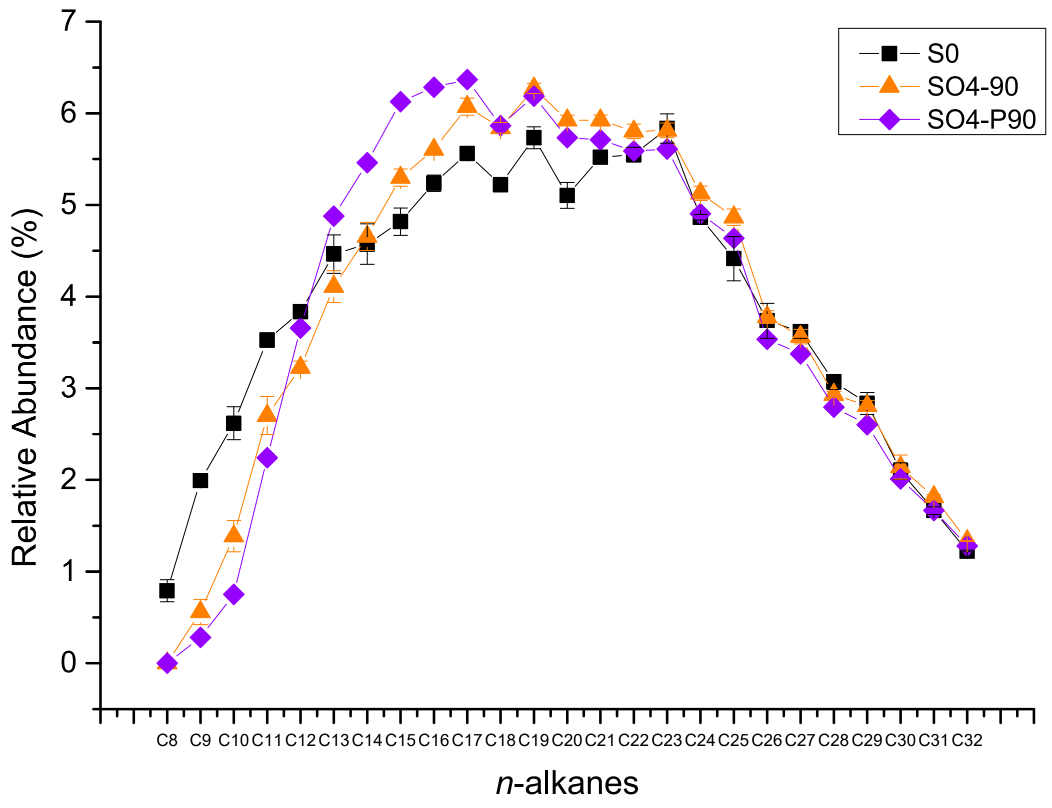

S0
SO4-90
SO4-P90
Relative Abundance (%)
0
1
2
3
4
5
6
7
C8
C9
C10
C11
C12
C13
C14
C15
C16
C17
C18
C19
C20
C21
C22
C23
C24
C25
C26
C27
C28
C29
C30
C31
C32
n-alkanes

Figure S3

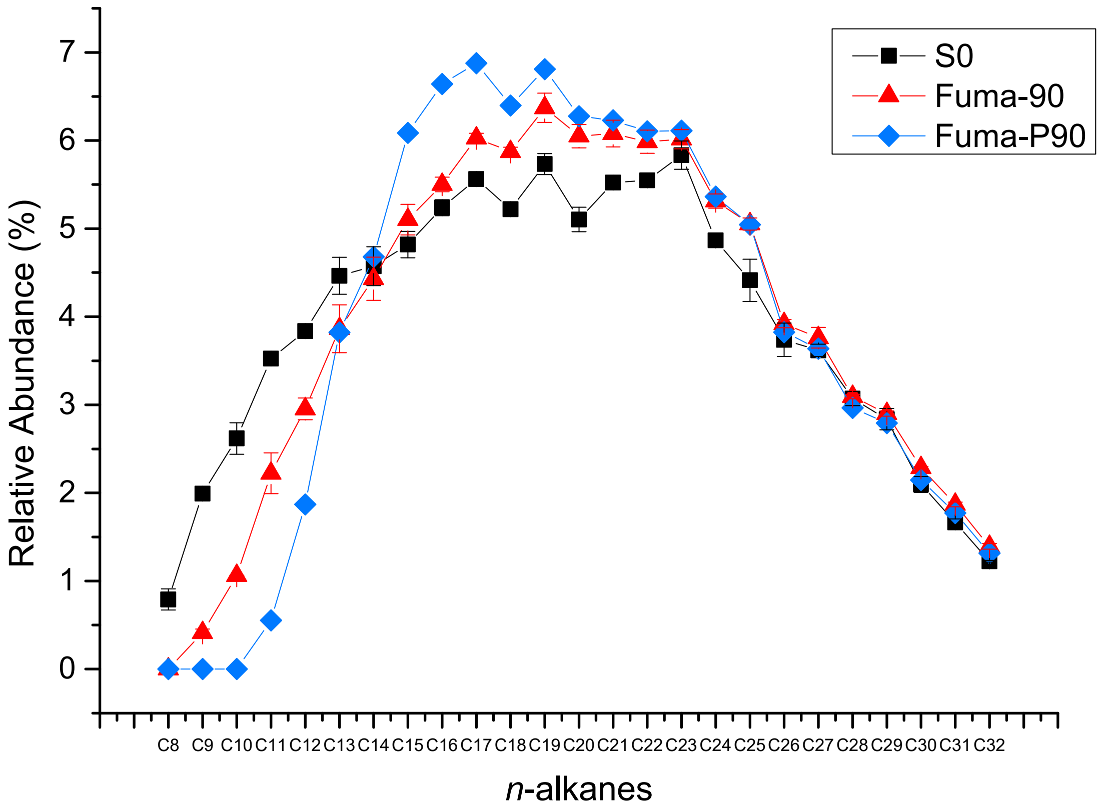

S0
Fuma-90
Fuma-P90
Relative Abundance (%)
7
6
5
4
3
2
1
0
C8 C9 C10 C11 C12 C13 C14 C15 C16 C17 C18 C19 C20 C21 C22 C23 C24 C25 C26 C27 C28 C29 C30 C31 C32
n-alkanes

Figure S4

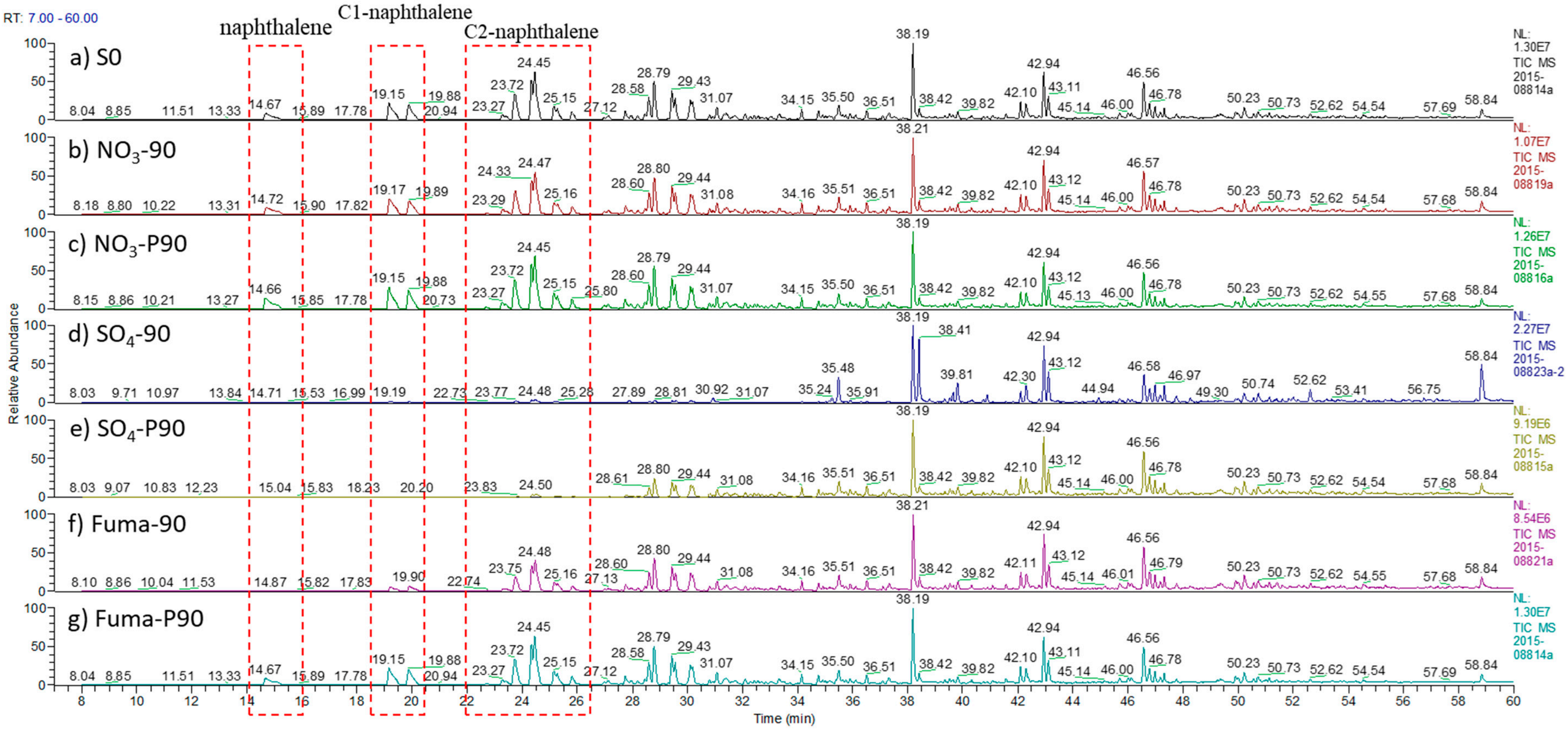
RT: 7.00 - 60.00
naphthalene
C1-naphthalene
C2-naphthalene
a) S0
b) NO3-90
c) NO3-P90
d) SO4-90
e) SO4-P90
f) Fuma-90
g) Fuma-P90
Relative Abundance
Time (min)

Figure S5
